# Synergic fabrication of pembrolizumab loaded doxorubicin incorporating microbubbles delivery for ultrasound contrast agents mediated anti-proliferation and apoptosis

**DOI:** 10.1080/10717544.2021.1921080

**Published:** 2021-07-14

**Authors:** Huilin Liu, Xing Li, Zihe Chen, Lianjie Bai, Ying Wang, Weiyang Lv

**Affiliations:** a Department of Ultrasound, The Second Affiliated Hospital of Qiqihar Medical College, Qiqihar City, PR China; b School of Medical Technology, Qiqihar Medical University, Qiqihar City, PR China

**Keywords:** Pembrolizumab, microbubble, ultrasound, theranostics, B cell lymphoma

## Abstract

This study evaluated pembrolizumab-conjugated, doxorubicin (DOX)-loaded microbubbles (PDMs) in combination with ultrasound (US) as molecular imaging agents for early diagnosis of B cell lymphomas, and as a targeted drug delivery system. Pembrolizumab, a monoclonal CD20 antibody, was attached to the surfaces of DOX-loaded microbubbles. PDM binding to B cell lymphoma cells was assessed using immunofluorescence. The cytotoxic effects of PDMs in combination with ultrasound (PDMs + US) were evaluated *in vitro* in CD20+ and CD20– cell lines, and its antitumor activities were assessed in Raji (CD20+) and Jurkat (CD20–) lymphoma cell-grafted mice. PDMs specifically bound to CD20+ cells *in vitro* and *in vivo*. Contrast enhancement was monitored *in vivo via* US. PDM peak intensities and contrast enhancement durations were higher in Raji than in Jurkat cell-grafted mice (*p* < 0.05). PDMs + US treatment resulted in improved antitumor effects and reduced systemic toxicity in Raji cell-grafted mice compared with other treatments (*p* < .05). Our results showed that PDMs + US enhanced tumor targeting, reduced systemic toxicity, and inhibited CD20+ B cell lymphoma growth *in vivo*. Targeted PDMs could be employed as US molecular imaging agents for early diagnosis, and are an effective targeted drug delivery system in combination with US for CD20+ B cell malignancy treatment.

## 1. Introduction

Early diagnosis is pivotal for therapeutic success in many types of cancers. Ultrasound (US) molecular imaging is a novel diagnostic approach for early detection of non-Hodgkin lymphoma (Wang et al., 2017; Heo et al., 2019; Toumia et al., 2019). Recent studies suggest that targeted microbubbles as US contrast agents (TMUCA) may serve as probes for US molecular imaging. TMUCA would improve diagnostic specificity and allow for disease monitoring in real time. TMUCAs can accumulate and remain at the tumor site for long time periods, and imaging at the molecular level can be acquired using US after TMUCA venous injection (Sanna et al., 2011; Li et al., 2018; Chen et al., 2019a,b; Zhang et al., 2020). US molecular imaging also produces quantitative data, exhibits good temporal resolution, is noninvasive, produces no ionizing radiation, and is relatively inexpensive. Over the past decade, various types of TMUCA have been applied for cell-specific targeting with US molecular imaging *in vivo*, specifically to assess intravascular inflammation, intravascular thrombosis, and tumor blood vessels (Chen and Sun, 2021; Zhu et al., 2021; Huang et al., 2021a,b). For early tumor diagnosis, TMUCAs were conjugated with antibodies specific for tumor cell surface antigens. Previous studies showed that the tumor neovasculature is distorted, with an imperfect basement membrane, and no smooth muscle layer. Permeability was also increased, with wall pores approximately 380–780 nm in diameter. Therefore, TMUCA diameters were adjusted to approximately 500 nm for easy passage through vascular endothelial cells and improved molecular imaging (Liu and Huang, 2011; Song et al., 2015; Wu et al., 2018; Zheng et al., 2021).

Targeted microbubbles are promising tumor-targeting drug delivery systems, although their potential utility as US contrast agents has not yet been studied. Most chemotherapy drugs currently have no targeting capabilities, and act on both diseased and non-diseased sites, leading to low therapeutic indices and severe side effects (Padmanabhan et al., 2016; Picheth et al., 2017; Guo et al., 2018; Paris et al., 2019; Wang et al., 2019). A targeted drug delivery system can increase chemotherapy drug accumulation specifically at target sites, while reducing non-target impacts (Ding et al., 2019; Li et al., 2020; Zheng et al., 2020; Feng et al., 2021; Chen et al., 2021; Wang et al., 2021; Ma et al., 2021). Moreover, targeted microbubbles are both chemically stable and biodegradable, and exhibit prolonged circulation in the blood, with localized drug release (Lux et al., 2017; Chen et al., 2019 a,b; Szablowski et al., 2019; Brambila et al., 2020). Tumor-specific ligand-like peptides, galactose-conjugated chitosan, transferrin, folic acid, and monoclonal antibodies have been employed to target microbubbles to tumor cells for the treatment of many cancers (Chertok et al., 2016; Chen et al., 2017; Tang et al., 2018). Additionally, the combination of targeted drug-loaded microbubbles with US irradiation permeabilizes cell membranes, enhancing drug uptake by tumor cells, and selectively killing tumor cells without harming normal cells. Therefore, targeted drug-loaded microbubbles have potential use in both targeted drug delivery systems and in combination with US molecular imaging (Kheirolomoom et al., 2010; Liu et al., 2021; Huang et al., 2021a,b).

We hypothesized that pembrolizumab-conjugated, doxorubicin (DOX)-loaded microbubbles (PDMs) could serve as effective, biocompatible B cell lymphoma-targeting theranostic agents. The present work evaluated the specific binding potential of PDMs targeting CD20 antigen, a tetraspan membrane receptor overexpressed in B cell malignancies, in lymphoma Raji cells. We also assessed the cytotoxicity and antitumor activity of these PDMs in combination with US irradiation *in vitro* and *in vivo*. Finally, targeted US molecular imaging was explored in Raji and Jurkat cell-grafted mice.

## 2. Materials and methods

### 2.1. Materials

Poly(lactic-co-glycolic acid) (PLGA; 50% lactide, 50% glycolide, MW = 10,000 Da) was purchased from Shandong Shuyuan Biotechnology Co., Ltd (Shandong, China). Poly(vinyl alcohol) (PVA, 87–89%, MW = 31,000–50,000) was obtained from Sigma-Aldrich (St Louis, MO). DOX was obtained from Shenzhen Wanle Pharmaceutical Co., Ltd. (Shenzhen, China). EZ-LinkTM Sulfo-LC-Biotinylation kit was purchased from Thermo Fisher Scientific, Inc. (Rockford, ILz. Amine-Peg2000-Biotin was purchased from Nanjing Ling Di Ren Chemical Technology Co., Ltd (Nanjing, China). Avidin and dyLight488-labeled avidin were obtained from Wuhan Boster Biotechnology Co., Ltd (Wuhan, China). Pembrolizumab was obtained from Hoffmann-La Roche, Inc (Little Falls, NJ). Cell Counting Kit-8, the Annexin V-FITC cell apoptosis detection kit, and the TUNEL apoptosis detection kit were purchased from Beyotime Biotechnology Co., Ltd (Shanghai, China). All chemicals were analytical grade and used without further processing.

### 2.2. DM preparation

PLGA microbubbles incorporating DOX were fabricated *via* a double US emulsion evaporation procedure. Of 0.5 g PLGA was fully dissolved in 10 mL of liquid chloroform *via* agitation. The PLGA solution was then combined with a 5 mg DOX solution (dissolved in 1.0 mL superpure water), and the mixture was emulsified *via* US for 120 min at 100 w. 1.0 mL span-80 was then added. The vial was degassed and re-perfused with nitrogen with stirring at 23,000 rpm for 5 min to obtain primary emulsified DMs. The primary emulsion was poured into cold PVA (40 mL, 5%) containing 1.0 mL tween-80, and stirred at 21,000 rpm for 30 min at room temperature for the second emulsion. The double emulsion was poured into isopropyl alcohol (40 mL, 2.5%) and mechanically agitated for 180 min at room temperature to volatilize the chloroform. The supernatant was removed after the solution was centrifuged at 4800 rpm for 5 min. The precipitate was centrifuged again at 1800 rpm for 5 min, and resuspended in superpure water. The superpure water wash was repeated several times until the supernatant become transparent. Precipites were resuspended a final time in superpure water and stored at 4 °C. DMs were sterilized *via* cobalt 60 (60Co) irradiation (Oliveira et al., 2019; Shi et al., 2020; Lainović et al., 2020; Xiao et al., 2020; Liu et al., 2021).

### 2.3. PDM preparation

Covalent bonding of the activated carboxyl groups on DM surfaces was performed using the 1-ethyl-3-[3-dimethylaminopropyl] carbodiimide hydrochloride (EDC) method in the presence of N-hydroxysuccinimide (NHS). Prepared DMs were resuspended in phosphate-buffered saline (PBS; pH 4.7), and EDC and NHS in an equimolar ratio were added into the suspension. The carboxyl groups were activated for 60 min at room temperature. The supernatant was removed after centrifugation, and the precipitate was resuspended in PBS. Amine-Peg2000-Biotin in MES buffer was added, and the mixture was incubated for 120 min at room temperature to obtain biotinylated DMs. Biotinylated DMs were incubated with avidin or dylight488-labeled avidin (1 mg/mL) for 10 min at room temperature. The mixture was then centrifuged three times and resuspended in PBS to remove surplus dylight488-labeled avidin/avidin. Pembrolizumab was biotinylated using the EZ-LinkTM Sulfo-LC-Biotinylatio kit according to the manufacturer’s instructions, and was added to the avidin-biotin conjugated DMs and incubated for 10 min. The PDM suspension was rinsed three times and centrifuged to remove surplus biotinylated pembrolizumab.

### 2.4. PDM characterization

We explored PDM morphologies using SEM (Hitachi S-3400N, Tokyo, Japan) and TEM (Hitachi H-7600, Tokyo, Japan) , and determined PDM mean diameters and size distributions *via* dynamic light scattering (DLS) (Nanosizer-S, Malvern, London, UK). PDMs were also observed using a CLSM (Olympus, FV1000, Tokyo, Japan). Pembrolizumab coupling efficiency was determined by measuring dyLight488-labeled avidin solution and biotinylated DMs suspension absorbances with a fluorescence spectrophotometer (Jasco, FP-6500, Tokyo, Japan) at a maximum excitation wavelength of 493 nm and maximum emission wavelength of 518 nm. Pembrolizumab quantities on biotinylated DMs (binding efficiency (%)) were calculated as the ratio of the intensity of biotinylated DM to the intensity of the dyLight488-labeled avidin samples.

### 2.5. Assessment of DOX loading

Drug encapsulation efficiency was assessed by ultraviolet-visible spectrophotometry (Eppendorf, BioSpectrometer, Hamburg, Germany). A DOX solution standard curve was measured. Then, fresh PDMs were centrifuged and collected. The PDMs were destroyed using a 5% hydrochloric acid ethanol solution, and the mixture was centrifuged at 3000 rpm for 5 min. The optical density of the supernatant was determined at an excitation wavelength of 495 nm. Drug encapsulation efficiency was calculated using the following equation: Encapsulation efficiency = Wa/Wb × 100%, where Wa represents the total amount of drug in the PDMs, and Wb represents the total weight of DOX used in the PDM preparation (Delplace et al., 2014; Deng et al., 2015; Kim et al., 2015; Parker et al., 2016).

### 2.6. Drug release assay

To estimate DOX release, PDM suspensions were enclosed in dialysis bags (MWCO: 10,000 Da), which were placed in 50 mL of PBS with shaking at 100 rpm at 37 °C. The suspension was then sonicated with US (power density = 1.2 W/cm^2^, frequency = 1 MHz, duty cycle = 50%) for 60 s. At 0, 2, 4, 8, 10, 20, 30, 48, 60, and 72 h, 1 mL of dialysate was extracted and stored at 4 °C for analysis. An equal volume of PBS was added to the container to insure a constant volume. The concentration of DOX in the sample was determined using an ultraviolet spectrophotometer. DOX release was depicted as a function of time. The DM suspension was assessed using the same method (Santha Moorthy et al., 2017; Zhang et al., 2018; Hu et al., 2019; Singh et al., 2019).

### 2.7. Cell cultures

Human lymphoma B cell lines Raji (CD20+) and Daudi (CD20+), human lymphoma T cell line Jurkat (CD20–), and human T-acute lymphoblastic leukemia cell line CEM (CD20–), were grown in RPMI-1640 medium with 10% (v/v) fetal bovine serum (FBS, Gibco, Waltham, MA, Australian origin) and 1% penicillin-streptomycin, and incubated in a humidified atmosphere at 37 °C with 5% CO_2_. For all experiments, cells growing in suspension were subcultured by centrifugation at a ratio of 1:4. After Raji and CEM cells were anchored in culture dishes with Poly L lysine solution, 50 uL targeted PDMs and non-targeted DMs were added into the dishes. Shaking was used to encourage interactions in Raji cell cultures. After 30 min at room temperature, dishes were washed twice with PBS and observed *via* CLSM. Five dishes were used for each experiment group. Blocking tests were performed by pre-incubating Raji cells with pembrolizumab for 30 min followed by washing to removing excessive pembrolizumab. CD20– CEM cells were employed as a control, and nonspecific uptake of PDMs by CEM cells was examined using the same methods.

### 2.8. Cytotoxicity *in vitro*

Raji, Daudi, Jurkat, and CEM cells were seeded in 96-well plates at 1 × 105 cells/well in 100 μL of RPMI-1640 medium. Microbubble samples were adjusted to contain equal amounts of DOX. Cells were treated with DOX (0.5 μg/mL final concentration), DOX + pembrolizumab, DMs combined with ultrasound (DMs + US), PDMs combined with ultrasound (PDMs + US), and PDMs + US. Raji cells were pre-incubated with excessive pembrolizumab for 30 min (PDMs + US + pembrolizumab). After 24, 48, and 72 h, the Cell Counting Kit-8 was used to detect viable cells in each treatment group relative to controls (Dilnawaz et al., 2010; Kang et al., 2016; Escudero-Duch et al., 2019; Sokolova et al., 2020).

### 2.9. Cell apoptosis *in vitro*

Apoptosis (early and late stage) was determined using an Annexin V/propidium iodide apoptosis kit and flow cytometry (BD Biosciences, San Jose, CA) with Cell Quest software. Raji, Daudi, Jurkat, and CEM cells, after various treatments for 24, 48, and 72 h, were rinsed twice with cold PBS and resuspended in 195 μL of binding buffer solution. Cells were stained with 5 μl FITC-labeled Annexin V and 10 μL propidium iodide for 20 min at room temperature in the dark. Cells treated with medium alone were used as a control (Mohamed Subarkhan et al., 2016; Subarkhan and Ramesh, 2016; Chung et al., 2017; Mohan et al., 2018; Mohamed Subarkhan et al., 2019; Sathiya Kamatchi et al., 2020).

### 2.10. DOX fluorescence intensity

Treatments were as follows: DOX, DOX + Pembrolizumab, DMs + US, PDMs + US, and PDMs + US + pembrolizumab at final DOX concentrations of 0.5 μg/mL. Raji, Daudi, Jurkat, and CEM cells were washed three times with cold PBS after 48 h treatment, centrifuged, and resuspended in 500 μL PBS. Cells treated with medium alone were used as controls. Intracellular DOX retention (red fluorescence) was examined using flow cytometry. Relative fluorescence intensity (RFI) was calculated as: FIexperiment/FIcontrol.

### 2.11. PDM-enhanced contrast ultrasound imaging *in vivo*

Cells were inoculated subcutaneously into the backs of five nude mice per cell type, with 6 × 107 Raji cells per mouse and 2 × 107 Jurkat per mouse. Imaging was performed using an iU22 ultrasound system (Phllips, Amsterdam, Netherland) with a 12 MHz US probe, 0.1 mechanical index, and 54% gain. Mice were anesthetized by injecting 10% hydral and fixed to entirely expose the tumor under the US probe. Non-targeted DMs were injected first for imaging studies in grafted mice. After the expurgation of non-targeted DMs, the same amount of targeted PDMs was injected. The process was monitored continuously by ultrasonography. US contrast data were quantified with PHILIPS QLab version 8.1 software . The arrival time, time to peak, peak intensity, and duration of contrast enhancement were determined. The Animal Ethics Commitment of the Southeast University approved all animal experiments.

### 2.12. *In vivo* antitumor activity

Raji and Jurkat cell-grafted mice were established additionally as described above. When lymphoma volume reached approximately 100 mm^3^, Raji and Jurkat cell-grafted mice were randomly divided into six groups (five mice per group), respectively: control group (saline), DOX, DOX + pembrolizumab, DMs + US, PDMs + US, and PDMs + US + pembrolizumab. Each mouse was treated with the appropriate formulation (3 mg/kg) three times per week. Lymphoma sizes in all mice were examined *via* digital caliper, and calculated using the equation: *V*_tumor_ = LW2/2 (L: tumor length, W: tumor width). Lymphoma volumes and mouse body weights were determined before every injection. After 21 d of treatment, all mice were sacrificed. Lymphomas were extracted and fixed with 4% paraformaldehyde. To detect cell apoptosis in lymphoma tissues, tissues were sliced into thin sections and stained with a TUNEL apoptosis detection kit. Samples were then stained with DAPI to visualize cell nuclei under a CLSM (Liu et al., 2011; Mohamed Kasim et al., 2018; Raudenska et al., 2019; Guo et al., 2019; Li and Gao, 2020; Abdelzaher et al., 2021).

### 2.13. Statistical analysis

All experiments were performed in triplicate. Data were presented as means ± standard deviation and analyzed using SPSS version 16.0 software (SPSS, Chicago, IL). Comparisons were performed using Student’s t-test. *p* < .05 was considered a significant difference.

## 3. Results and discussion

### 3.1. PDM characterization

**Figure 1.**
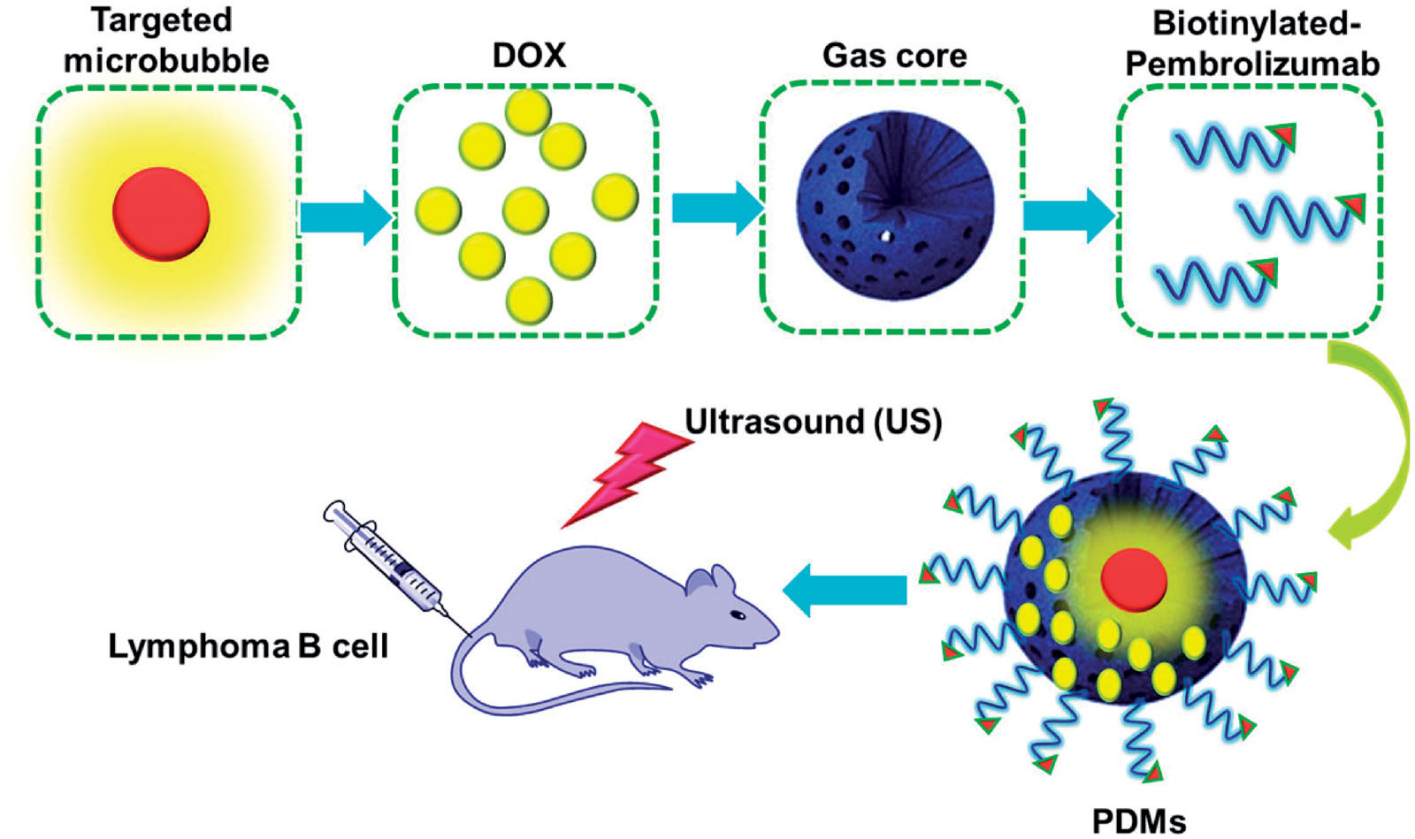
Illustration of PDM structure, and depiction of antigen-specific, tumor cell targeting drug delivery. After PDM attachment to CD 20 antigens on Raji cells, US irradiation triggers DOX release and delivery into cells. DOX: doxorubicin; PDMs: pembrolizumab-conjugated; DOX-loaded microbubbles.

Microbubble-based targeted drug delivery has been widely investigated as an anti-tumor therapy in combination with US irradiation. Most targeted drug delivery systems exhibit high therapeutic efficacies *in vitro* and *in vivo*. However, few studies have assessed their potential roles, and the roles of microbubbles, in ultrasonic molecular imaging for diagnosis. In this study, PDMs targeted the lymphoma B cell CD20 antigen. US triggered DOX release, which was then delivered into lymphoma B cells (Figure 1).

**Figure 2.**
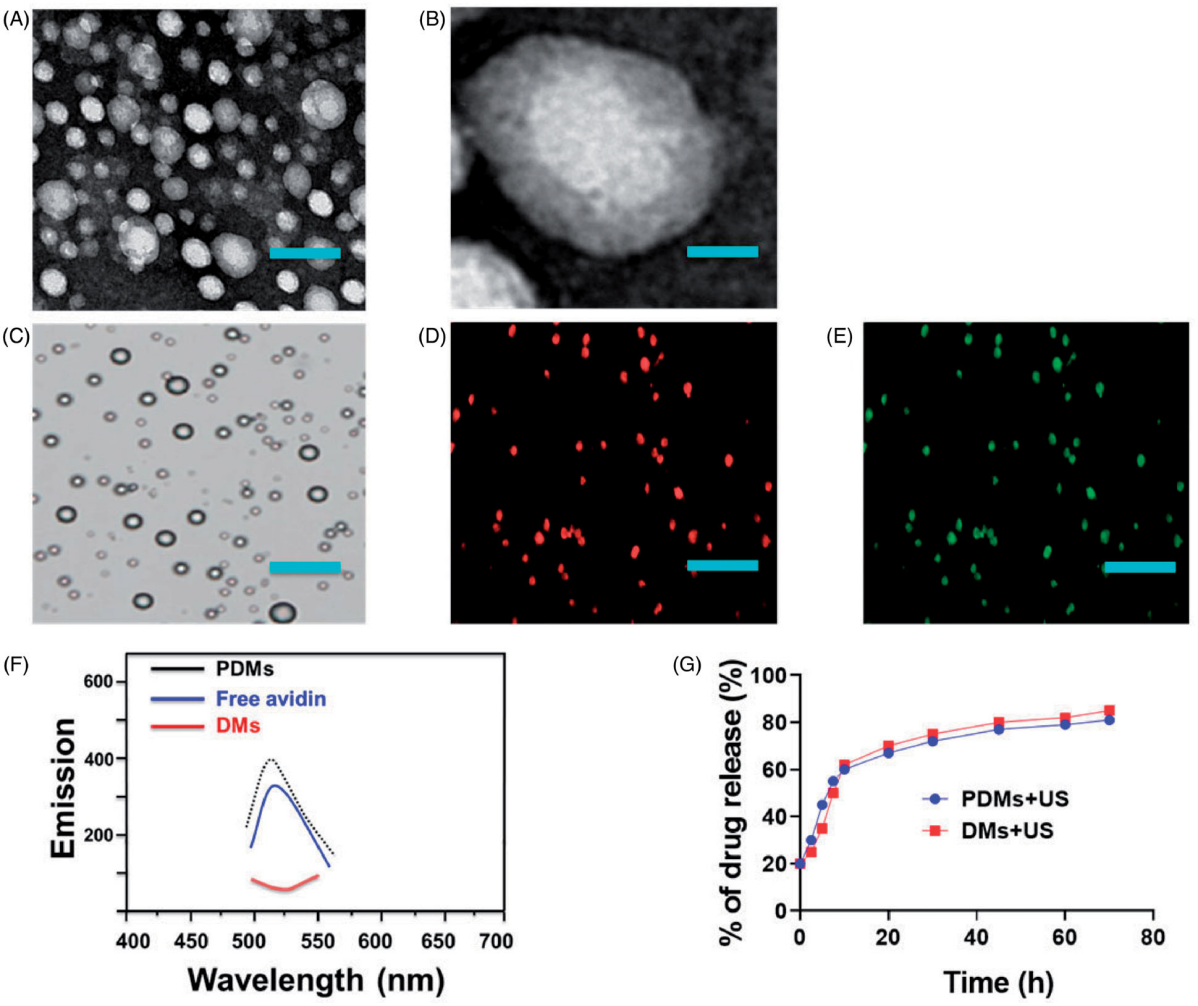
Microbubble characterization. A) SEM and B) TEM images of PDMs. Microbubble fluorescence and drug release characterization. C–E) PDM imaging was performed using CLSM as follows: bright field Aa., dylight488-avidin on PDMs (green fluorescence) Ab., encapsulated DOX in PDMs (red fluorescence) Ac. (scale bar = 25 μm). F) Fluorescence absorbance of PDMs. DMs and free avidin were examined to assess pembrolizumab conjugation efficiency. G) *In vitro* US-triggered DOX release from DMs and PDMs.

PDM morphologies and size distributions were observed *via* scanning electron microscopy (SEM) (Figure 2(A,B)) and transmission electron microscopy (TEM) (Figure 2(C–E)), respectively. Fluorescence imaging of PDMs revealed dense green (DyLight488-labeled avidin) and red (DOX) fluorescence with morphologies consistent with those observed *via* confocal laser scanning microscopy (CLSM) (Figure 2(D,E)). Targeting moiety quantities on PDM surfaces were evaluated by detecting PDM suspension fluorescence intensity after conjugation. PDM fluorescence intensity was 72.15% that of the DyLight488-labeled avidin samples (Figure 2(F)). Due to the high affinity of avidin to biotin, we presumed the same high level of adhesion of biotinylated pembrolizumab to the avidin-conjugated DMs.

### 3.2. Drug loading and release

PDMs as a targeted drug delivery system were evaluated *via* the encapsulation efficiency of DOX. DOX encapsulation efficiency in PDMs was 51.2 ± 2.05%. The release profiles of DOX from PDMs and DMs as triggered by US were also examined. The release profile was described as the percentage of cumulative released DOX as a function of time (Figure 2(G)). Total DOX released was the same for DMs + US and PDMs + US. The results indicated that DOX was about 50% unloaded after 5 h with sonication and about 90% unloaded after 72 h with sonication. This implied that US could promote DOX release from PDMs and DMs through cavitation.

### 3.3. Targeted properties of PDMs

**Figure 3.**
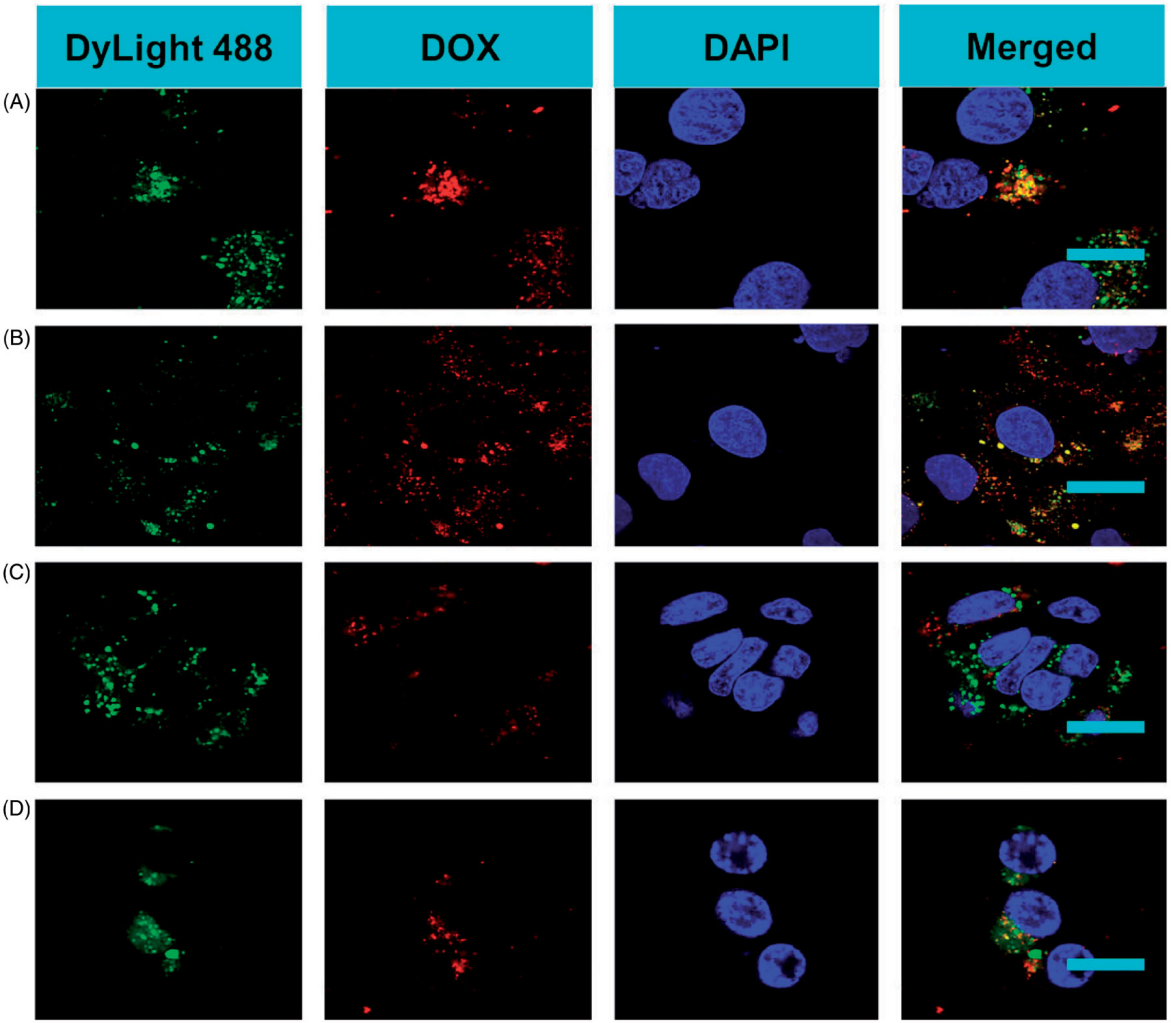
*In vitro* PDM targeting to Raji cells. CLSM imaging of Raji cells after treatment with targeted PDMs and non-targeted DMs. To visualize microbubble location (green and red fluorescence), Raji cell nuclei were stained with DAPI (blue fluorescence) (scale bar = 10 μm). A) Raji cells targeted by PDMs. B) Raji cells incubated with DMs. C) Raji cells blocked with excess Pembrolizumab (1 mg/mL) for 2 h before PDM treatment D) CEM cells incubated with PDMs as a control.

To estimate the targeted binding capability of PDMs, the affinity of PDMs to CD20 antigen on Raji cells was determined *in vitro*. PDM attachment to CD20 antigen was greater than that of DMs. CLSM imaging showed large amounts of PDMs (green and red fluorescence) aggregated on Raji cell membranes, demonstrating that pembrolizumab enhanced PDM targeted binding to CD20 antigen. Few DyLight488-labeled avidin-conjugated biotinylated DMs were observed on Raji cell membranes. Competition experiments revealed that PDM targeted binding of Raji cells was reduced as CD20 antigen was blocked following pembrolizumab pre-incubation, as indicated by absence of red and green fluorescent microbubbles. Few PDMs were observed on CD20– CEM cell membranes (Figure 3).

### 3.4. Cytotoxicity *in vitro*

**Figure 4.**
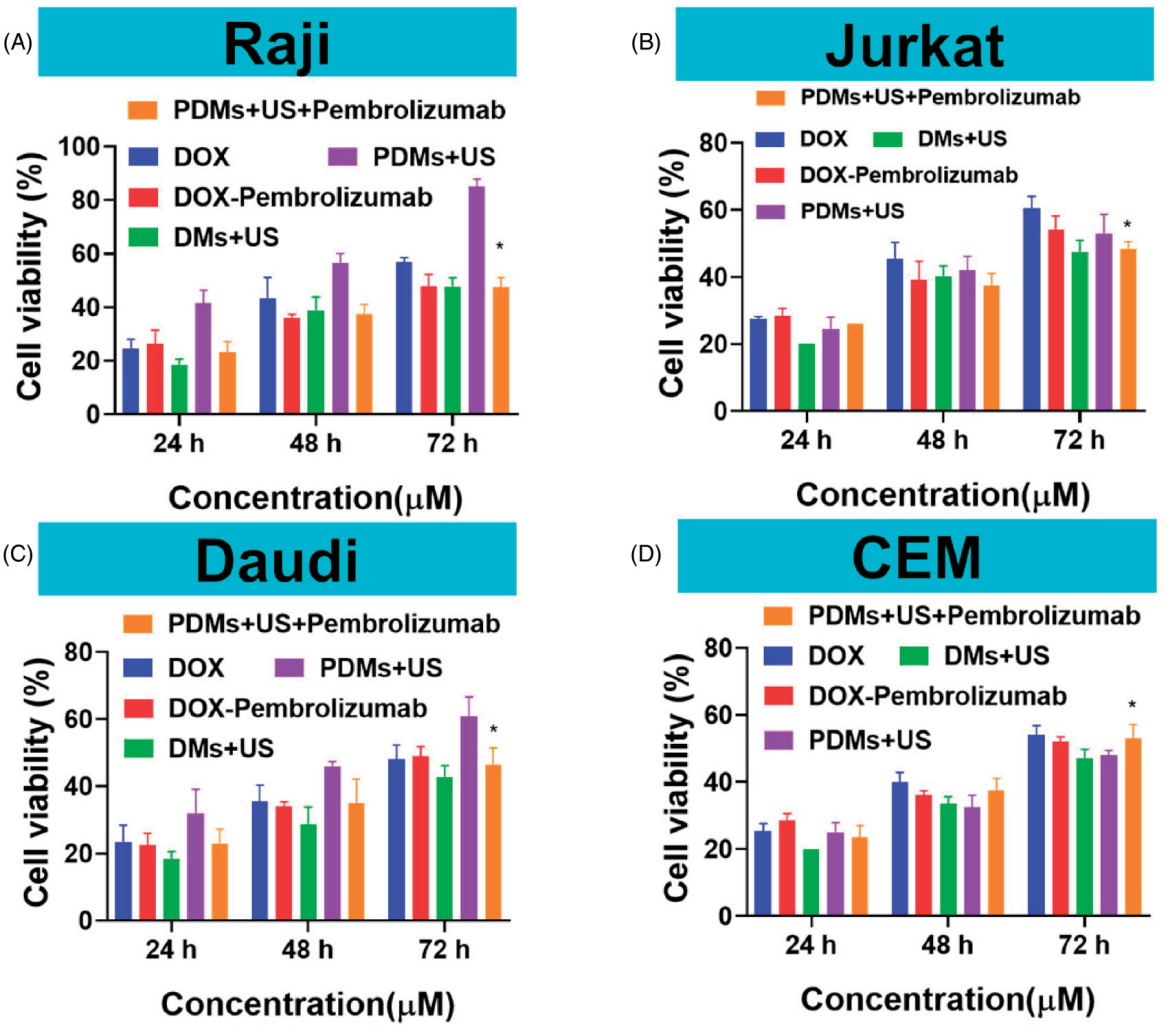
Raji, Daudi, Jurkat, and CEM cell growth inhibition. Cell proliferation inhibition was measured 24, 48, and 72 h after treatment with DOX, DOX + pembrolizumab, DMs + US, PDMs + US, and PDMs + US + embrolizumab *via* CCK8 assay. Data are represented as means ± SD (*n* = 3). *p < .05 compared with PDM + US.

The therapeutic efficacies of DOX, DOX + pembrolizumab, DMs + US, PDMs + US, and PDMs + US + pembrolizumab were explored *in vitro*. Raji, Daudi, Jurkat, and CEM cell proliferation inhibition was assessed after treatment for 24, 48, and 72 h. Raji and Daudi cell proliferation inhibition was limited, and was the same for DOX, DOX + pembrolizumab, DMs + US, and PDMs + US + pembrolizumab. In contrast, PDM + US inhibited Raji cell proliferation after 24 (35.42 ± 2.16%), 48 (52.32 ± 3.42%), and 72 h (82.74 ± 2.97%) (Figure 4(A)). PDM + US in Daudi cells also reduced proliferation at 24 (32.84 ± 3.31%), 48 (48.79 ± 2.71%), and 72 h (74.85 ± 3.52%) (Figure 4(C)). Compared with other treatments, PDM + US effectively inhibited Raji and Daudi cell proliferation (*p* < .05). However, in Jurkat and CEM cells, all treatments exhibited similar cytotoxicity (Figure 4(B,D)). PDM + US inhibited proliferation in Raji and Daudi cells more than in Jurkat and CEM cells (*p* < .05). We also observed time-dependent cytotoxic effects in all groups. These results demonstrated that US-mediated PDM destruction efficiently inhibited CD20+ lymphoma B cell proliferation.

### 3.5. Cell apoptosis *in vitro*

**Figure 5.**
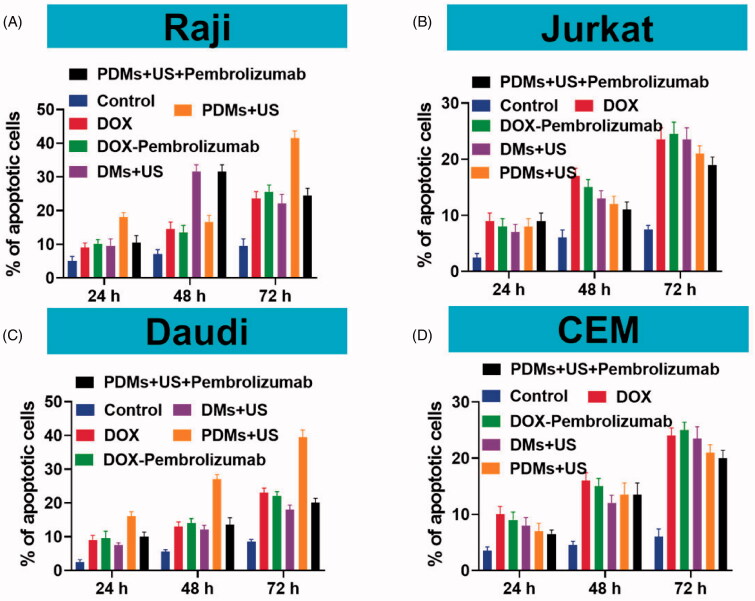
Raji, Daudi, Jurkat, and CEM cell apoptosis. Cell apoptosis was measured 24, 48, and 72 h after treatment with DOX, DOX + pembrolizumab, DMs + US, PDMs + US, and PDMs + US + pembrolizumab by flow cytometry. Data are represented as means ± SD (*n* = 3). *p < .05 compared with PDM + US.

Raji, Daudi, Jurkat, and CEM cell apoptosis rates were detected quantitatively by flow cytometry 24, 48, and 72 h after various treatments. Raji and Daudi cell apoptosis rates were comparable following DOX, DOX + pembrolizumab, DMs + US, and PDMs + US + pembrolizumab treatment, although apoptosis was increased in all groups compared to controls. Importantly, PDMs + US induced higher apoptosis rates than other treatments (Figure 5(A,C), *p* < .05). Jurkat and CEM cell apoptosis rates were similar for DOX, DOX + pembrolizumab, DMs + US, and PDMs + US + pembrolizumab, but higher compared to controls (Figure 5(B,D)). Additionally, PDM + US induced higher apoptosis rates in Raji and Daudi cells as compared to Jurkat and CEM cells (*p* < .05). Time-dependent apoptosis rates were detected in all groups (Figure 5). Apoptosis rate measurements were consistent with proliferation inhibition results.

### 3.6. Cellular uptake of DOX

**Figure 6.**
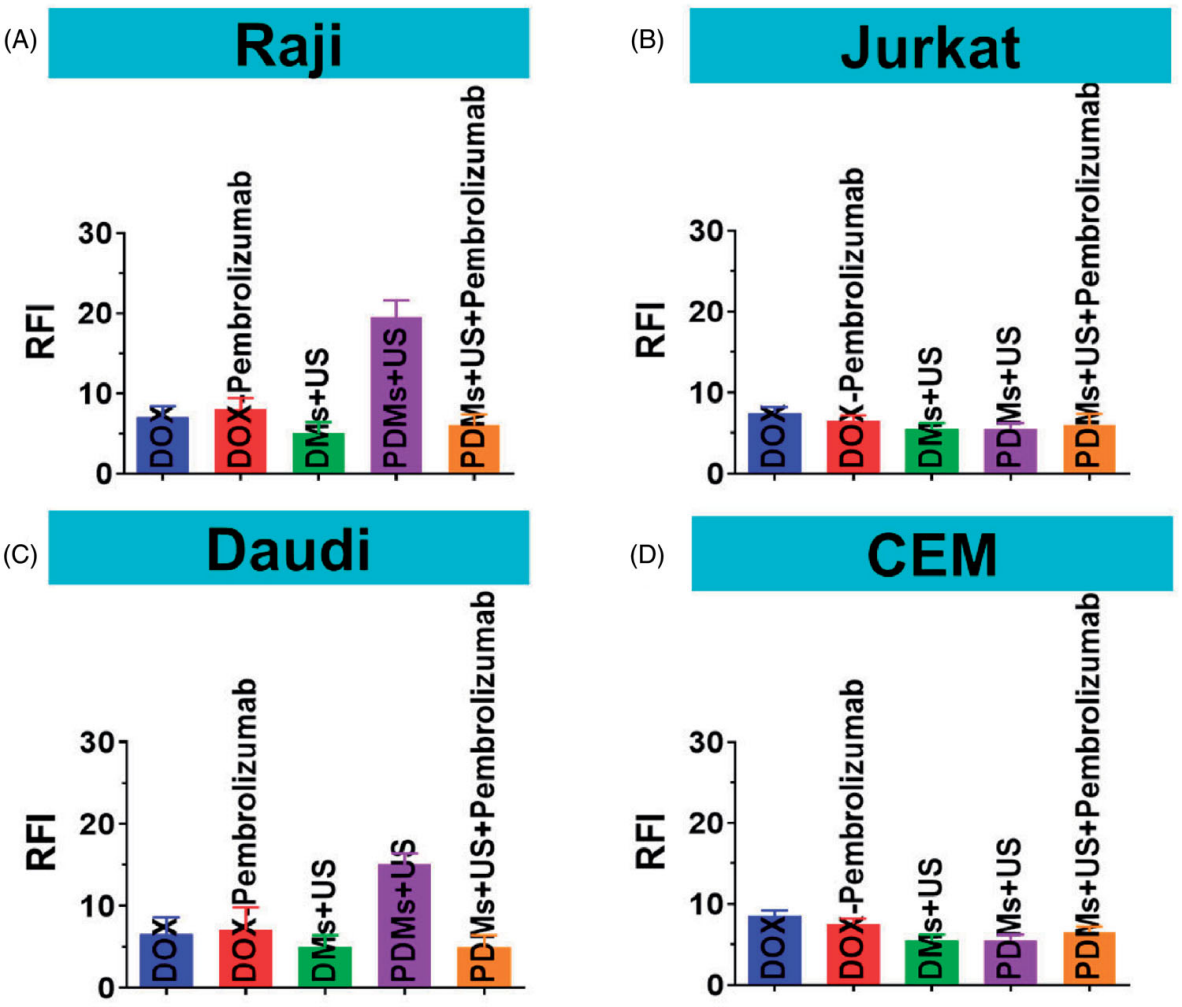
DOX relative fluorescence intensity (RFI) in Raji, Daudi, Jurkat, and CEM cells. Cell RFIs were analyzed 48 h after treatment with DOX, DOX + pembrolizumab, DMs + US, PDMs + US, and PDMs + US + pembrolizumab by flow cytometry. Data are represented as means ± SD (*n* = 3). **p < .01 compared with PDM + US.

Because DOX auto-fluoresces, we detected whether 48 h PDMs + US treatment improved Raji, Daudi, Jurkat, and CEM cell DOX uptake using flow cytometry. DOX intracellular RFI for Raji and Daudi cells was similar following DOX, DOX + pembrolizumab, DMs + US, and PDM + US + pembrolizumab treatment. However, DOX intracellular RFI following PDM + US was higher compared to all other treatments (Figure 6(A,C), *p* < .01). DOX intracellular RFI was also similar following all treatments in Jurkat and CEM cells (Figure 6(B,D)). Additionally, DOX intracellular RFI following PDM + US treatment was higher in Raji and Daudi cells as compared to Jurkat and CEM cells (*p* < .01). These results suggest that PDM + US treatment increased DOX transfer into lymphoma B cells more than other treatment groups.

### 3.7. *In vivo* imaging

**Figure 7.**
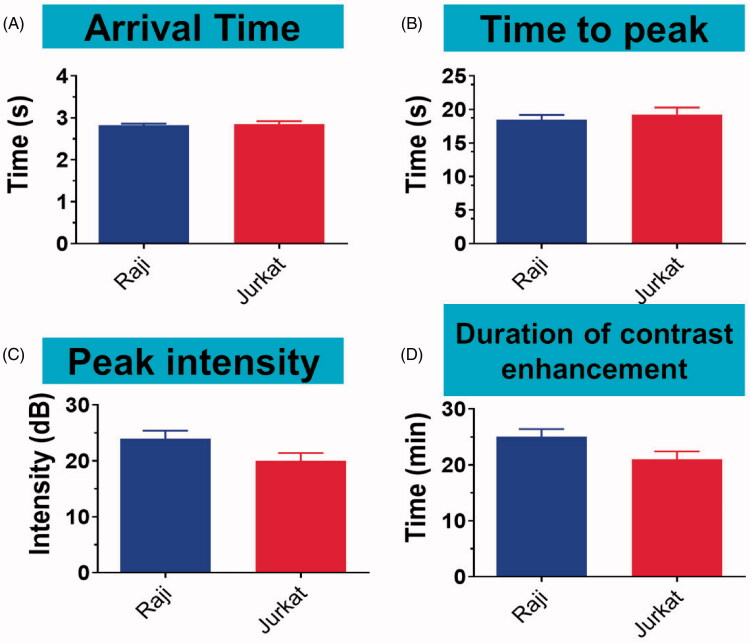
PDM arrival time, time to peak, peak intensity, and duration of contrast enhancement in Raji and Jurkat cell-grafted mice. PDM arrival times and times to peak were the same in Raji and Jurkat cell-grafted mice. PDM peak intensities and contrast enhancement durations were greater in Raji cell-grafted mice than in Jurkat cell-grafted mice. Data are represented as means ± SD (*n* = 3). *p < .05, ^#^*p* > .05.

**Figure 8.**
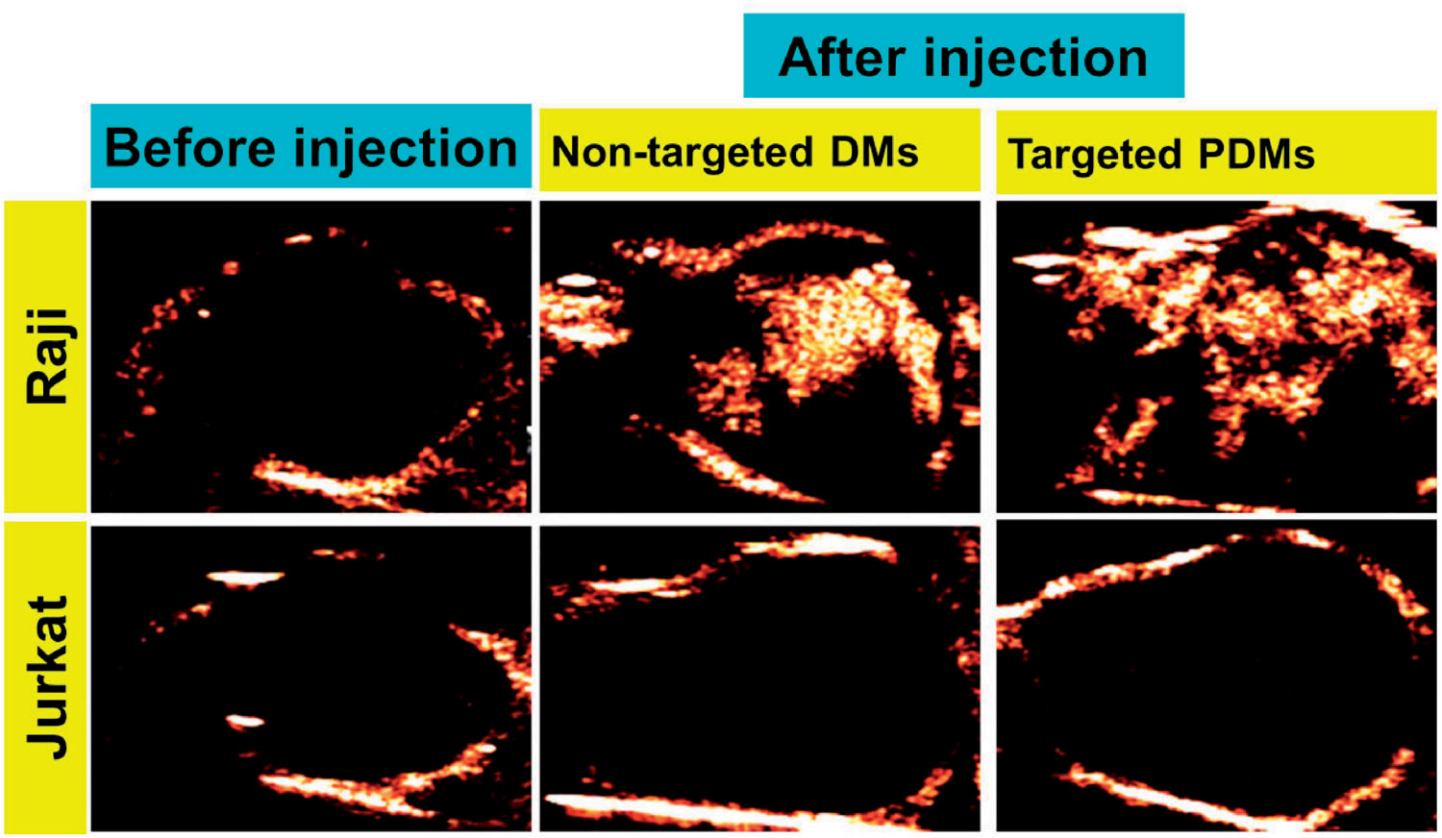
Contrast-enhanced images of targeted PDMs and non-targeted DMs at time to peak in Raji and Jurkat cell-grafted mice. Images of lymphoma before injection A and D, non-targeted DMs B and E, and targeted PDMs C and F at time to peak were acquired in Raji and Jurkat cell-grafted mice. PDM peak intensities and contrast enhancement durations were higher than those of non-targeted DMs in Raji cell-grafted mice, and were higher in Raji as compared to Jurkat cell-grafted mice.

Arrival time, time to peak, peak intensity, and duration of contrast enhancement were compared *via* US imaging between non-targeted DMs and targeted PDMs in Raji and Jurkat cell-grafted mice. In Raji cell-grafted mice, there was no difference between DMs and PDMs for arrival time or time to peak, but peak intensity and duration of contrast enhancement were higher for PDMs (*p* < .05). In Jurkat cell-grafted mice, there was no difference between DMs and PDMs in any US measurement. Additionally, arrival times and times to peak for targeted PDMs were the same in Raji and Jurkat cell-grafted mice. However, PDM peak intensities and the durations of contrast enhancement were higher in Raji as compared to Jurkat cell-grafted mice (Figure 7, *p < .05). Targeted PDM (Figure 8(C,F)) and non-targeted DM (Figure 8(B,E)) peak intensity images are shown for Raji and Jurkat cell-grafted mice.

### 3.8. *In vivo* antitumor activity

**Figure 9.**
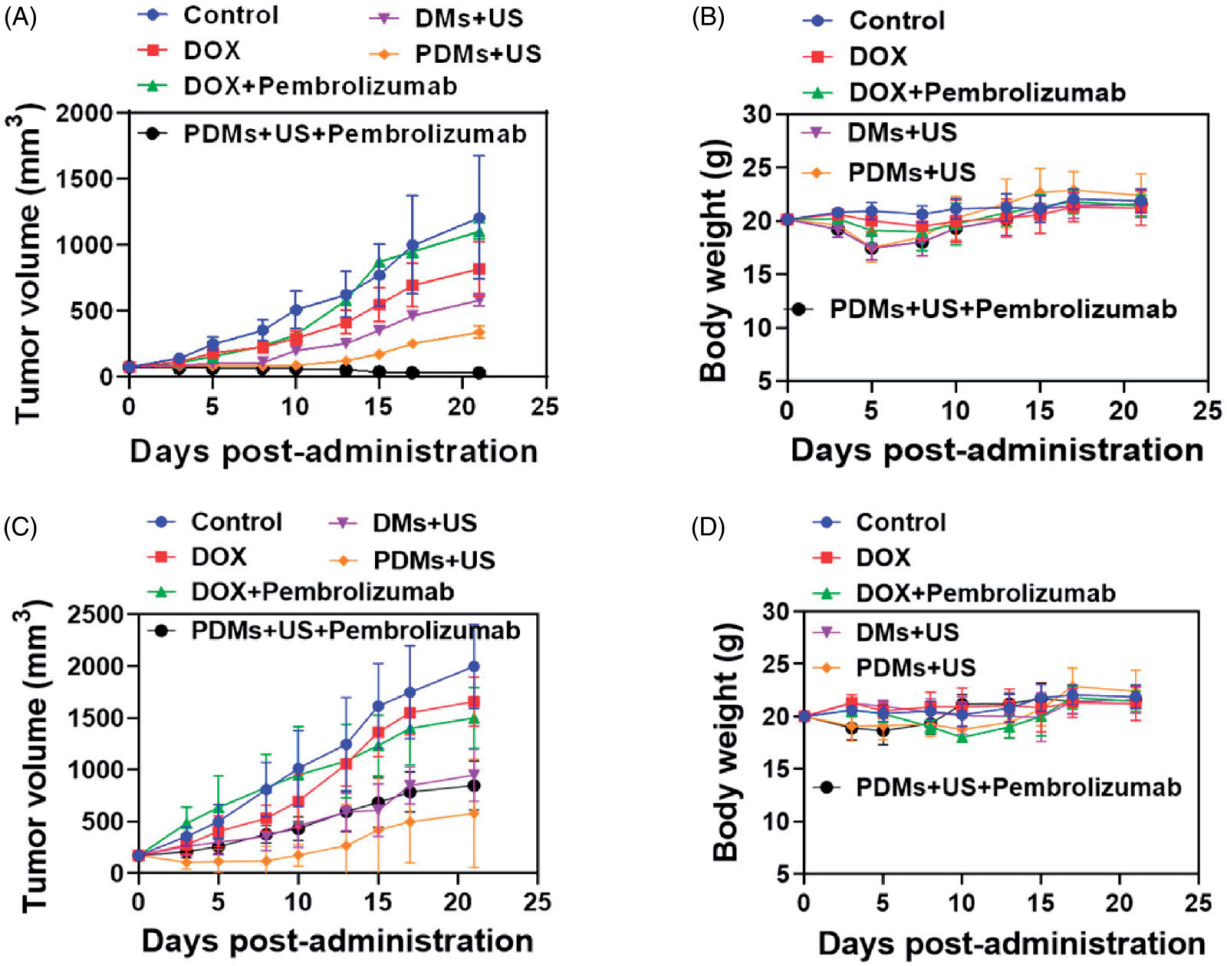
Anti-tumor treatment effects in Raji and Jurkat cell-grafted mice. Average tumor volumes A. and body weights B. of Raji cell-grafted mice after treatment with different formulations. Average tumor volumes C. and body weights D. of Jurkat cell-grafted mice after treatment. Data are represented as means ± SD (*n* = 3). *p < .05.

This study used a lymphoma nude mouse model to investigate the antitumor effects of PDMs + US *in vivo*. PDMs + US exhibited the strongest tumor inhibition effect in Raji-cell grafted mice. DMs + US, and PDMs + US + pembrolizumab-treated mice exhibited similarly reduced Raji cell tumor growth rates compared to controls, and inhibited tumor growth more than treatment with DOX and DOX + pembrolizumab (Figure 9(A)). DOX and DOX + pembrolizumab only slightly inhibited tumor growth *in vivo*. Similarly, DMs + US, PDMs + US, and PDMs + US + pembrolizumab treatment reduced grafted Jurkat cell tumor growth as compared with DOX and DOX + pembrolizumab. Jurkat cell-grafted mouse treatment with DMs + US, PDMs + US, and PDMs + US + pembrolizumab resulted in comparable growth inhibition rates (Figure 9(C)).

To assess the potential systemic toxicity of PDMs + US *in vivo*, nude mouse body weights were periodically examined. Raji and Jurkat cell-grafted mice treated with DOX and DOX + Pembrolizumab exhibited slow, continuous weight loss beginning on day 8. In contrast both Raji and Jurkat cell-grafted mouse weights increased gradually with saline, DMs + US, PDMs + US, and PDMs + US + pembrolizumab treatments (Figure 9(B,D)). This suggests that DOX treatment caused severe systemic toxicity in nude mice.

**Figure 10.**
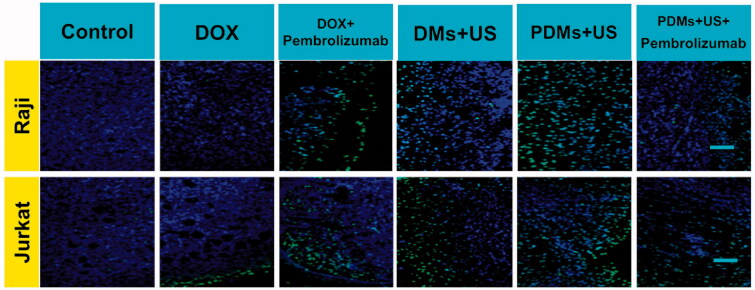
TUNEL staining of Raji and Jurkat lymphomas treated with different formulations. Raji and Jurkat cell-grafted mice were treated with saline, DOX, DOX + pembrolizumab, DMs + US, PDMs + US, and PDMs + US + pembrolizumab for 21 d. Green: apoptotic cell DNA; Blue: cell nuclei. Scale bar: 100 μm.

TUNEL staining was used to evaluate apoptosis in Raji and Jurkat cell tumors. Sparse apoptosis (green fluorescence) in Raji and Jurkat lymphoma tissues was observed in mice treated with DOX and DOX + pembrolizumab. Raji cell-grafted mouse tissues treated with DMs + US, and PDMs + US + pembrolizumab showed moderate apoptosis, while PDMs + US treatment induced the most apoptosis. Jurkat cell-grafted mouse tissues treated with DMs + US, PDMs + US, and PDMs + US + pembrolizumab showed moderate cell apoptosis. We observed that PDMs + US induced greater apoptosis levels in Raji as compared to Jurkat cell-grafted mice (Figure 10). These results confirmed that PDMs + US could inhibit lymphoma cell growth by inducing apoptosis.

## 4. Conclusion

In conclusion, this study indicated that targeted PDMs specifically bound CD20+ B cell lymphomas. PDMs combined with US irradiation enhanced tumor targeting, reduced systemic toxicity, and inhibited B cell lymphoma cell growth *in vivo*. Additionally, targeted PDMs increased peak intensity and contrast enhancement duration compared to non-targeted DMs in CD20+ B cell lymphoma-grafted mice. Our findings show that targeted PDMs could potentially be employed as US molecular imaging agents for early diagnosis, and are an effective targeted drug delivery system in combination with US irradiation for the treatment of CD20+ B cell malignancies.
